# Intravenous flurbiprofen axetil can increase analgesic effect in refractory cancer pain

**DOI:** 10.1186/1756-9966-28-33

**Published:** 2009-03-07

**Authors:** Hongyang Wu, Zhendong Chen, Guoping Sun, Kangsheng Gu, Yueyin Pan, Jiqing Hao, Yingying Du, Jie Ning

**Affiliations:** 1 Department of Medical Oncology, The First Affiliated Hospital of Anhui Medical University, Hefei, 230022, PR China

## Abstract

**Background:**

The aim of this study was to investigate the analgesic effects of intravenous flurbiprofen axetil for the refractory pain in cancer patients.

**Methods:**

2109 patients were screened from the department of medical oncology, the first affiliated hospital of Anhui medical university in China between October of 2007 and October of 2008. Thirty-seven cases of cancer patients who had bad effect from anaesthetic drugs were received administration of intravenous flurbiprofen axetil with dose of 50 mg/5 ml/day. The pain score was evaluated for pre- and post- treatment by Pain Faces Scale criteria, and the side effects were also observed.

**Results:**

Intravenous flurbiprofen axetil increased the analgesic effects. The total effective rate was 92%. The side effects, such as abdominal pain, alimentary tract bleeding which were found in using NSAIDs or constipation, nausea, vomit, sleepiness which were found in using opioid drugs did not be found.

**Conclusion:**

Intravenous flurbiprofen axetil could provide better analgesia effects and few side effects to patients with refractory cancer pain. It could also increase analgesia effects when combining with anesthetic drugs in treatment of moderate or severe pain, especially breakthrough pain, and suit to patients who can not take oral drugs for the reason of constipation and psychosomatic symptoms.

## Background

Pain is a common problem in cancer patient. About one-third of patients being treated for cancer have pain. More than two-thirds of patients with advanced cancer have pain [1]. Controlling pain and managing symptoms are important goals of cancer treatment [2].

Flurbiprofen is a non-selective cyclooxygenase inhibitor used in clinic as nonsteroidal anti-inflammatory drug [3]. Flurbiprofen axetil, an injectable prodrug of flurbiprofen [4], has been reported to be associated with a reduction postoperative pain [5,6], propofol injection pain [7,8], and in initial treated pain induced from cancer [9]. The role of flurbiprofen axetil are not yet clear in the routinely administration of refractory cancer pain. In the present study, we reported the role of intravenous flurbiprofen axetil in this area.

## Methods

### Patients

Cancer pain cases whose pain had not been treated satisfactorily with routine narcotics were selected from the department of medical oncology, the first affiliated hospital of Anhui medical university in China between October of 2007 and October of 2008. Each cancer case was diagnosed and confirmed by histopathology or cytopathology. Clinical data and follow-up information were obtained from the hospital records. The study protocol was approved by the local institutional ethics committee, and verbal informed consent was obtained from each patient. Patients with difficulty communicating, a history of adverse response to flubiprofen axetil, or who felt no pain after received other analgesic drugs within 24 hours were excluded.

### Dosage and usage of flurbiprofen axetil injection

All selected patients were received 50 mg/5 ml/day of intravenous flurbiprofen axetil injection (50 mg/5 ml, Beijing Tide Pharmaceutical. Co., Ltd, Beijing, China), as flurbiprofen axetil 50 mg added in 100 ml of 0.9% isotonic saline every time through vein within 30 minutes. Dosage and usage of the anaesthetic drugs such as Oxycodone, Tramadol, Duragesic and adjuvant drugs such as diazepam, carbamazepine which being used initially were not changed, or be reduced and ceased after the pain was relieved completely. Other accompanying adjuvant treatments also had been included chemotherapy, radiotherapy, best sustain therapy, bisphosphonate therapy, and etc.

### Evaluating criteria

We evaluated cancer pain intensity by Pain Faces Scale criteria [10], and the three grades as: Mild pain (1–3): Cancer pain could be endurable, and sleep was effected slightly, action was freely, no pain was in the patient's face; Moderate pain (4–6): Cancer pain could be endurable yet, and sleep was effected obviously, action was limited, pain was showed in the patient's face; Severe pain (7–10): Cancer pain could not be endurable, and sleep was effected severely, action was limited hardly, more pain was showed in the patient's face, body's style was passively.

Pain relief was recorded based on four-scale criteria [11]: Complete relief (CR): The pain was disappeared completely, or alleviated significantly, no anesthetic drug must be used; Partial relief (PR): The pain was alleviated significantly than before. The sleep was not disturbed by and large. Patient could live in normal or use a few anesthetic drugs; Minimal relief (MR): The pain was alleviated than before, but it still felt obviously. The sleep was still disturbed by the pain, and the dosage of anesthetic drugs was not reduced significantly than before; No effect (NR): The pain was not alleviated significantly than before, or the dosage of anesthetic drugs were not reduced than before.

CR and PR were regarded as effective response to cancer pain treatment.

### Side effects

Side effects were observed and classified according to the WHO acute and sub-acute toxicity classifying criteria of anticancer drugs [12]. Some symptoms such as swirl, nausea, vomit, abdominal pain, diarrhea, astriction, dysuria, vessel stimulate, etc, were noticed especially after flurbiprofen axetil had being used.

## Results

A total of 2109 patients were screened. 37 patients were enrolled based on the criteria (22 men, 15 women; mean [SD] age, 57[13] years, mean [SD] height, 161[9] cm; mean [SD] body weight, 56[11] kg). Other clinic characteristics of those patients were showed in Table 1.

Thirty-three cases of refractory cancer pain were received 50 mg of intravenous flurbiprofen axetil injection every day. Other four cases had to increase the dosage of flurbiprofen axetil to 100 mg a day for the reason of insufficient effect by 50 mg a day. Thirty-four patients were regarded as partial relief or complete relief. The total effective rate was 92%. The results of usage and analgesic effect were showed in Table 2.

The side effect, gastrointestinal toxicity such as abdominal pain, alimentary tract ulcers and bleeding which were found in NSAIDs or constipation, nausea, vomit, sleepiness and delirium which were found in opioid drugs did not be found in all of the 37 cancer pain cases.

**Table 1:** Clinical characteristics of 37 patients with refractory cancer pain (number)

**Cancer stage**	**number**
III stage	2
IV stage	35
**Primary cancer**	
gastric (cardia)	5
oesophageal	1
rectal	1
lung	18
breast	3
prostate	3
the primary site not clear	6
**Pain reason**	
bone metastasis	33 (including one incomplete ileus)
pleura invasion	2
ileus	2
**Pain intensity**	
moderate	26
severe	11

**Table 2:** The usage and analgesic effect of flurbiprofen axetil in refractory cancer pain (number)

**Using time (day)**	
Short	2
Long	34
average	12.5
mean	7
**The initially anaesthetic drugs (number)**	
dosage and usage not changed	20
dosage decreased slightly	8
dosage decreased significantly	6
the initially drugs ceased	3
**Combining with treatment (number)**	
chemotherapy	23
radiotherapy	2
best sustain therapy	6
bisphosphonate therapy	10
**Pain relief (number)**	
complete relief	10 (9, bone metastasis; 1, pleura metastasis)
partial relief	24 (bone metastasis)
minimal relief	3 (2, abdominal pain in gastric cancer; 1, pleura aggression of lung cancer)

## Discussion

In this study, we testified that intravenous flurbiprofen axetil injection could increase the analgesic effects in refractory cancer pain. Lipid microspheres (LM) were target-drug delivery carriers which could congregate selectively in the site such as inflammation or injuring blood vessel and change the distribution of drugs in vivo [13,14]. Flurbiprofen axetil injection, 0.2 μm in diameter, was composed of lipid microspheres and flurbiprofen axetil[15]. It was target-congregated easily to tumor, especially malignant tumor for there had abundant fresh capillary vessel and released inflammatory factor. The latter could enlarge the fissure of endothelium cells and let it be taken up by macrophages and neutrophils. So, the biosynthesis of prostaglandin was restrained, and the analgesic effects of flurbiprofen axetil would be appeared [16]. Flurbiprofen axetil injection always had better analgesic effects in bone metastasis of tumor while nociceptor pain was mainly expressed [9].

Anaesthetic anodynes were always used in moderate and severe pain patients. It acted in central nerve system, and the analgesic effects was not relative with the site or kind of pain. But, side effects always happened, such as constipation, breath inhibition, drug dependence, even exciting central nerve system when it was used for long time [2]. Flurbiprofen axetil and other NSAIDs drugs acted in the site of distal nerve. Its analgesic effects were always not bad than anaesthetic anodynes when the inflammatory medium was liberated in the site of muscle, tendon, ligament, and bone. It could be used as first line anodyne and combined with anaesthetic anodynes in corresponding cancer pain [4]. Our results showed that intravenous flurbiprofen axetil had better analgesic effect to cancer pain with bone or vertebra metastasis. It could reduce the dosage of the anaesthetic drugs, or increase the analgesic effects with little side effect, especially in patient who had constipation or had a tendency of ileus. Our results showed the analgesic effect was better than the Ou Yang's report [9], and similar to the report by Xu et al [17]. Perhaps for the reason of insufficient cases, we found that flurbiprofen axetil had slight analgesic effect to cancer pain in abdomen.

The half-life time of flurbiprofen axetil was 5.8 hours. Its onset of action was about 15 minutes after being used, and continued about 3 hours in post-operation. When it was used in cancer patients, it began to work quickly about in 30 minutes, and the duration of action was about 9 hours [18]. So it was especially suitable for breakthrough pain to the patient who were using anaesthetic anodyne. We found that most patients could obtain analgesic effects after being added flurbiprofen axetil 50 mg while their pain could not be controlled by anaesthetic drugs. But in some patients, the analgesic effect was only maintained 3–4 hours. So it need to be used continually or time after time, or other analgetic administrations should be used to the patients whose had severe cancer pain but could not deal with mainline.

Sometimes oral NSAIDs drugs are restrictedly applied mainly for the reason to stimulate patient's gastric mucosa. Intravenous flurbiprofen axetil injection could avoid this side effect. In all of 1089 cases, the side effect incidence rate was very low about 2.9% [18]. Most side effects were in gastrointestinal tract such as nausea, vomit, diarrhoea or in neuropsychosis such as fever, fear cold, sleepiness, etc. Few cases expressed as subcutaneous bleeding or pain in the injecting site. Perhaps our cases were insufficient, no side effect of flurbiprofen axetil was found in this study.

## Conclusion

In general, cancer pain is considered as chronic. The pain intensity ranges from mild to severe and present for a long time. Harmless approach to therapy such as by oral or by cutaneous are suggested by WHO. But, for some reasons as constipation and psychosomatic symptoms, there has many patients whose can not take drugs by oral, or can not be used cutaneous anaesthetic drugs, intravenous flurbiprofen axetil could exactly remedy the anaesthetic drug's shortcoming, and let itself to be an important switch drug.

## Competing interests

The authors declare that they have no competing interests.

## Authors' contributions

HW collected the data and drafted the manuscript, ZC designed this study and modified the manuscript, GS, KG, YP, JH, YD, JN participated in its design and coordination. All authors read and approved the final manuscript.
